# Associations between dairy food consumption and chronic kidney disease in older adults

**DOI:** 10.1038/srep39532

**Published:** 2016-12-20

**Authors:** Bamini Gopinath, David C. Harris, Victoria M. Flood, George Burlutsky, Paul Mitchell

**Affiliations:** 1 Centre for Vision Research, The Westmead Institute for Medical Research, University of Sydney, NSW, Australia; 2 Centre for Transplantation and Renal Research, The Westmead Institute for Medical Research, University of Sydney, NSW, Australia; 3 Faculty of Health Sciences, University of Sydney and St Vincent’s Hospital, Australia

## Abstract

We aimed to assess the association between dairy product consumption and calcium intake with the prevalence and 10-year incidence of chronic kidney disease (CKD). 1185 participants aged ≥50 years at baseline were examined between 1992–4 and 2002–4. Dietary data were collected using a food frequency questionnaire, and servings of dairy food consumption were calculated. Baseline biochemistry including serum creatinine was measured. CKD was defined as Modification of Diet in Renal Disease Study estimated glomerular filtration rate <60 mL·min^−1.^1.73 m^−2^. Cross-sectional analysis showed that older adults in the highest quintile compared to the lowest quintile (reference group) of low/reduced fat dairy food consumption had reduced odds of CKD, multivariable-adjusted odds ratio, OR, 0.64 (95% confidence intervals, CI, 0.43–0.96). Increasing total intake of dietary calcium was associated with reduced odds of CKD (*P*-trend = 0.02); comparing highest versus lowest quintile: OR 0.62 (95% CI 0.42–0.92). Participants in the second versus first quintile of low/reduced fat dairy food consumption at baseline had 49% reduced risk of CKD 10 years later, OR 0.51 (95% CI 0.29–0.89). Higher consumption of low/reduced fat dairy foods was independently associated with lower risk of CKD. Additional population-based studies are warranted to confirm these findings.

There is accumulating evidence from published studies showing habitual consumption of dairy foods could counteract risk of obesity, metabolic syndrome, type 2 diabetes, hypertension and cardiovascular disease^1^,^2^,^3^,^4^. Conversely an inadequate intake of dairy foods and calcium has been linked with reductions in bone mass and increased risk of osteoporosis^5^, increased risk of age-related macular degeneration^6^, poorer retinal microvascular profile^7^ and lower levels of adiponectin (an anti-inflammatory cytokine)^8^. Given that systemic risk factors such as obesity, diabetes, and hypertension have also been linked with chronic kidney disease (CKD)^9^, there is a potential for regular dairy product consumption to modify renal function in older adults. Moreover, regular consumption of dairy products has been demonstrated to confer protection against inflammation, oxidative stress and endothelial dysfunction^3^,^10^. Given that the published literature indicates that increased oxidative stress and inflammation may mediate some of the effects of risk factors on renal dysfunction^11^,^12^ these could be potential pathways by which dairy foods could modify the risk of developing CKD. However, there are a lack of studies that have examined the link between dairy intake and CKD risk. One of the few studies to have recently examined this association was the Atherosclerosis Risk in Communities (ARIC) Study, which specifically analysed the link between the Dietary Approaches to Stop Hypertension (DASH) diet score and risk of CKD^13^. This study showed that of the individual components of the DASH diet score, low-fat dairy product intake was associated with reduced risk for kidney disease.

We previously showed in a cohort of older Australians aged 50 years and over that individual nutritional parameters could influence the prevalence and incidence of CKD^14^. Specifically, a novel link was established between high cereal fiber intake and reduced incidence of CKD, which was supported by a cross-sectional association with dietary glycemic index^15^. Further, we showed that increased dietary intake of long-chain omega-3 and fish also reduced the prevalence of CKD in this cohort^14^,^16^. We have, however, yet to examine whether dairy foods have a role to play in the development of CKD among older adults. Given that CKD is a growing public health problem globally, with rising incidence and prevalence;^17^ it is imperative to comprehensively investigate lifestyle modifications such as weight reduction, exercise and dietary manipulations which could be potentially effective and protective against this chronic disease^18^.

Therefore, in the current cohort study of older adults, we aimed to answer the following key questions: 1) Is regular consumption of dairy products (comprising of the three primary dairy foods consumed in our study population-milk, cheese, and yoghurt) and total calcium intake associated with the prevalence and 10-year incidence of CKD, independent of potential confounders such as age, sex, body mass, smoking, serum triglycerides, hypertension and diabetes? and 2) Do any associations found differ by the type of dairy foods consumed i.e. regular/high fat compared to low/reduced fat dairy products?

## Methods

### Study population

The current report uses data collected in the Blue Mountains Eye Study (BMES), a population-based study of participants aged ≥49 years, living in two postcodes of the Blue Mountains region, west of Sydney, Australia, which has studied age-related eye diseases and other health outcomes in an older urban Australian population. Details of the study methods have previously been described^19^. In brief, during 1992–4, 3654 participants 49 years or older were examined (82.4% participation; BMES-1). Surviving baseline participants were invited to attend 5-year follow-up examinations (1997–9, BMES-2), at which 2335 (75.1% of survivors) were examined. At the 10-year follow-up (2002–4, BMES-3), 1952 participants (75.6% of survivors) were re-examined. The study was approved by the Human Research Ethics Committee of the University of Sydney and was conducted adhering to the tenets of the Helsinki Declaration. Signed informed consent was obtained from all participants at each examination.

### Assessment of dairy consumption

Dietary data were collected using a 145-item self-administered food frequency questionnaire (FFQ), modified for Australian diet and vernacular from an early Willett FFQ^20^ and including reference portion sizes. Participants used a 9-category frequency scale to indicate the usual frequency of consuming individual food items during the past year. The FFQ was validated by comparing nutrients from the FFQ to 3 × 4-day weighed food records collected over one year (n = 79) in order to assess seasonal variation. Most nutrient correlations were between 0.50 and 0.60 (e.g. 0.61 for calcium) for energy-adjusted intakes, similar to other validated FFQ studies^21^,^22^. A dietician coded data from the FFQ into a customized database that incorporated the Australian Tables of Food Composition 1990 (NUTTAB 90) and follow-up data used NUTTAB95^23^,^24^.

Dairy sub-categorization includes regular milk, reduced fat/skim milk, low fat cheese, regular cheese, reduced fat dairy dessert (e.g. low fat yoghurt) and medium fat dairy dessert (e.g. custard and regular yoghurt). Total dairy included all of the aforementioned dairy foods; low/reduced fat dairy included ‘reduced fat/skim milk’, ‘reduced fat dairy dessert’ and ‘low fat cheese’; while regular fat dairy included ‘regular milk’, ‘regular cheese’ and ‘medium fat dairy dessert’. Quintiles of dairy product consumption were based on servings of dairy consumed/day. The serving sizes used were 250 g for milk, 200 g for yoghurt, 250 g for custards, and 40 g for cheeses^1^. Total calcium intake was assessed from foods consumed only and did not include calcium intake from dietary supplements. We also examined whether meeting the recommended daily intake for total dairy foods (2.5 serves/day or more) and calcium intake (1300 mg/day or more) in Australian adults^25^ influenced the likelihood of having CKD in our cohort.

### Assessment of CKD

Serum creatinine was measured within 4 hours of fasting venous blood collection using a Hitachi 747 biochemistry analyzer (Roche Diagnostics, Castle Hill, Sydney, NSW, Australia). Serum creatinine data from 2003–4 (BMES-3) was measured in an Isotope Dilution Mass Spectrometry (IDMS) aligned version of the assay. However, BMES-1 serum creatinine is not IDMS aligned. Therefore, we applied a correction to BMES-1 serum creatinine data to bring the assay results into alignment with IDMS. The approach taken is to use the known conversion factors supplied by Roche from the Roche Jaffe rate blanked uncorrected assay and the same assay with correction. The “Correction” referred to is to bring the assay into alignment with IDMS. The correction is a mathematical one based on value assignment of the calibrators and subtraction of a factor. BMES-1 creatinine data have been converted to IDMS equivalent results using the formula: Creatinine (IDMS) = Creatinine (BMES-1) × 1.086 −26 μmol/L.

Estimated GFR (eGFR) was the preferred measure of kidney function in the current study. GFR was indirectly estimated using the 4-variable Modification of Diet in Renal Disease Study (MDRD) equation: GFR (mL·min^−1.^1.73 m^−2^) = 186 × (serum creatinine)^−1.154^ × (age)^−0.203^ × (0.742 if female) (conventional units)^26^. The main outcome of interest was CKD of stage 3 or greater, defined as an eGFR of <60 mL·min^−1.^1.73 m^−2^.

### Assessment of covariates

At face-to-face interviews with trained interviewers, a comprehensive medical history that included information about demographic factors, socio-economic characteristics (including whether participants were receiving a pension or not) and lifestyle factors such as smoking, was obtained from all participants. History of smoking was defined as never, past, or current smoking. Current smokers included those who had stopped smoking within the past year. Diabetes was defined either from history or by fasting blood glucose ≥7.0 mmol/L. Subjects were defined as having hypertension if they had systolic blood pressure greater than 140 mm Hg or diastolic blood pressure more than 90 mm Hg or were on anti-hypertensive medications^27^. Body mass index (BMI) was calculated as weight divided by height squared (kg/m^2^). Total serum triglycerides were measured on a Reflotoron reflectance photometric analyzer (Roche Diagnostics, Castle Hill, Sydney, NSW, Australia).

### Statistical analysis

SAS statistical software (SAS Institute, Cary NC) version 9.2 was used for analyses including t-tests, χ^2^-tests and logistic regression. Associations between quintiles of dairy food consumption (including regular fat and low/reduced fat dairy products) and prevalence and 10-year incidence of CKD (study outcome) were examined in logistic regression models, adjusted first for age and sex, and then further adjusting for confounders that were significantly associated with CKD (i.e. receipt of pension, smoking, BMI, history of diabetes mellitus, hypertension, serum triglycerides), in the BMES cohort. Given the overlap in regular-fat and low-fat dairy product consumption among BMES participants^6^, all analyses were adjusted for high-fat dairy intake when the study factor was low-fat dairy intake, and if the study factor was high fat dairy food intake then low-fat dairy intake was included in the multivariable model. Correlation coefficient between change in regular fat dairy and low-fat dairy product intake over 15-years was moderate i.e. −0.34 (p < 0.0001), hence, it was appropriate to adjust for both regular-fat and low-fat dairy product consumption in the final, multivariable model. The logistic regression analyses are expressed as adjusted odds ratios (OR) with 95% confidence intervals (CI).

## Results

Of the 3654 participants at baseline, 2689 participants had complete serum creatinine and FFQ data, and hence, were included for cross-sectional analyses. Table 1 shows a comparison of baseline characteristics between persons with and without CKD. Participants with versus those without CKD were older, less likely to be male and to smoke, but more likely to be on a pension, have higher serum triglycerides, and also more likely to give history of hypertension, and diabetes. Table 2 shows the association between dairy food consumption and prevalence of CKD. Participants in the fourth versus first quintile (reference group) of total dairy food intake had 36% reduced odds of CKD after adjusting for all potential confounders. Participants in the highest quintile compared to the lowest quintile of low/reduced fat dairy food intake had 36% reduced likelihood of CKD (Table 2). No significant associations were observed with the consumption of high/regular dairy food consumption.

Of the 2689 participants examined at baseline 730 had died over the 10 years and 1952 were survivors (there were 7 people with missing mortality information). Participants who died versus those who survived were older, more likely to be male, a smoker, receive a pension, and have hypertension, diabetes and a lower BMI (Online Supplementary Table). Those who died were also more likely to have lower intakes of total dairy, low fat dairy and dietary calcium compared to survivors. Of the surviving 1952 individuals re-examined at the 10-year follow-up, 612 did not have serum creatinine data at both baseline and follow-up, and a further 155 did not have dietary data at baseline, hence, these participants were excluded from incidence analyses leaving 1185 participants. Study characteristics of participants who had serum creatinine data compared to those who did not have this data were compared. We found that participants without creatinine data compared to those with this data had lower intake of reduced/low fat dairy foods (p = 0.01), no other significant differences in baseline characteristics were observed between these two groups. Table 3 shows that participants in the second versus the first (lowest) quintile of low/reduced fat dairy food consumption at baseline had 49% reduced risk of developing CKD 10 years later, multivariable-adjusted OR 0.51 (95% CI 0.29–0.89). Non-significant associations were observed between total dairy intake and regular/high fat dairy food consumption and 10-year incidence of CKD.

We also assessed whether total dietary intake of calcium (mg/day) influences risk of CKD. Table 4 presents cross-sectional analysis and shows that increasing total intake of dietary calcium was associated with reduced odds of CKD (*P*-trend = 0.02), comparing highest versus lowest quintile: multivariable-adjusted OR 0.62 (95% CI 0.42–0.92). Increasing intake of total dietary calcium was not associated with the 10-year incidence of CKD (*P*-trend = 0.26).

Supplementary analysis involved assessing the association between meeting the recommended daily intake for total dairy foods (2.5 serves/day or more) and calcium intake (1300 mg/day or more) with both the prevalence and incidence of CKD. At baseline, 24.3% and 16.6% of participants met the recommended daily intake of dairy foods and calcium, respectively. No significant associations were observed between meeting compared to not meeting the recommended daily intake of dairy and calcium and prevalence of CKD: OR 0.94 (95% CI 0.71–1.24) and OR 0.85 (95% CI 0.61–1.19), respectively. Similarly, meeting the recommended daily intake for dairy products and calcium compared to not meeting these recommendations were also not associated with incident CKD: OR 0.78 (95% CI 0.53–1.16) and OR 0.70 (95% CI 0.44–1.10), respectively.

## Discussion

This community-based cohort of older adults provides unique epidemiological data showing that habitual consumption of low/reduced fat dairy foods among older adults was independently associated with reduced prevalence and incidence of CKD. Further, total dietary calcium was inversely associated with the prevalence of CKD but not with the 10-year incidence of CKD. Consumption of total dairy foods and regular/high fat dairy products did not influence the risk of CKD in the longer-term.

Our finding that consumption of low/reduced fat dairy foods was inversely associated with the prevalence and incidence of CKD concurs with recent findings from the ARIC study, which also showed a protective effect of low-fat dairy products on CKD^13^. Further, previous studies have shown a protective effect of habitual dairy product consumption on various health outcomes including diabetes, hypertension and cardiovascular disease^1^,^2^,^3^,^4^ and this supports the protective effect of dairy foods observed in this cohort. Moreover, there is evidence to show that higher dairy product intake suppresses oxidative stress and inflammation among adults who are overweight and obese^28^. Given that there is evidence for oxidative stress and inflammatory processes in CKD^11^,^12^; the protection conferred by dairy foods against the development of CKD could be at least partly explained by the anti-inflammatory properties of dairy foods^11^,^12^. Despite the plausible explanation for the beneficial influence of dairy consumption on renal function, we advise caution because of the large number of comparisons made in this study, and hence, cannot disregard the possibility that the observed association could be due to chance.

Moreover, the presence of albuminuria is linked to CKD^29^ and it has been demonstrated that the presence of albuminuria predicts progressive CKD, independent of eGFR level^30^. A cross-sectional multiethnic study^31^ found that low-fat dairy food intake was inversely associated with the odds of microalbuminuria; conversely, high fat dairy food intakes were positively associated with albuminuria. The key constituents of most dairy products could underlie the observed beneficial effect on chronic conditions such as CKD, including: calcium, vitamin D, magnesium, and dairy proteins^3^. There is evidence to suggest that vitamin D (a micronutrient found in dairy foods) could also have a protective influence on renal function^32^. Although, it is widely recognized that almost a third of Australian adults suffer from a vitamin D deficiency^33^, we highlight that vitamin D levels were not measured in our cohort, and therefore, it is outside the scope of this study to explore whether vitamin D mediates the link between dairy foods and CKD. Further, in our study, total calcium intake over was inversely associated with the prevalence of CKD. This is biologically plausible as published literature shows that high calcium diets can suppress systemic oxidative and inflammatory stress^34^. Nevertheless, it is important to note that most participants in our cohort (83.4%) were not meeting the recommended daily intake of calcium (1300 mg/day)^25^,^32^.

Microvascular abnormalities in the kidney are common histopathologic findings in patients with CKD^35^. In the BMES, we previously showed that participants who did not consume any low-fat dairy products during the week (in the lowest quintile) had a poorer retinal microvascular profile as evidenced by both narrower retinal arteriolar caliber and wider retinal venular caliber. Additionally, lower total calcium intake was also associated with wider retinal venules among older adults^7^. Therefore, the influence of dairy food consumption and calcium intake could be operating, albeit partially, via a microvascular pathway. Nevertheless, we need to highlight that the BMES is an observational study and hence, we cannot confer a cause and effect relationship between dairy food consumption and risk of CKD. Further, prospective studies are needed to confirm our findings.

Nevertheless, our study concurs with most dietary guidelines that recommend consuming dairy foods in the low-fat form, and with studies that show low-fat dairy products to be protective against chronic diseases, including vascular disease^1^,^2^,^3^,^4^. Specifically, epidemiological data form the current study show that a diet incorporating adequate intakes of low-fat dairy foods and dietary calcium could have a protective influence on kidney function. Hence, our study is an important contribution to the existing literature as aging population trends in Western countries emphasizes the critical need for identification and development of potential protective strategies for CKD^16^.

Strengths of this study include its representative population-based sample with relatively high participation, minimizing selection bias, and use of a validated food questionnaire to collect dietary data and availability of rich covariate information. Limitations also deserve discussion. First, we need to caution that there were some inconsistencies between the cross-sectional and longitudinal findings. Specifically, a significant protective effect of low fat dairy consumption on prevalence of CKD was observed among participants in the highest quintile, however, a protective effect of low fat dairy foods against incident CKD was only observed in participants in the second quintile of consumption versus the first quintile. The small number of incident CKD cases, resulting in lack of statistical power could partly explain the discrepancy in findings. Second, residual confounding by other unknown or unmeasured parameters is also plausible but is speculative based on our known knowledge of risk factors for CKD prevalence and incidence in the BMES. Third, there were significant differences in study characteristics including in dietary intakes and clinical parameters (e.g. hypertension, diabetes etc.) between survivors and those participants who died over the 10-year period; hence, we cannot disregard selection bias influencing observed associations in this study. Finally, in the current study we only assessed calcium intake from foods consumed and did not include calcium intake from dietary supplements, hence, our observation that dietary calcium intake was inversely associated with the prevalence of CKD needs to be interpreted with caution.

In summary, we demonstrate that a higher consumption of low fat dairy foods could contribute to a modest decrease in the prevalence and incidence of CKD over a 10-year follow-up period. The BMES is an observational study and we cannot prove a cause-effect link, however, it does highlight a necessity to maintain adequate consumption of low-fat dairy foods with age^6^. Additional longitudinal studies of the association between dairy product consumption and risk of CKD are nevertheless required. Further research is also warranted to establish the causal pathway(s) that underlie the ability of dairy products to protect renal function in older adults.

## Additional Information

**How to cite this article**: Gopinath, B. *et al*. Associations between dairy food consumption and chronic kidney disease in older adults. *Sci. Rep.* **6**, 39532; doi: 10.1038/srep39532 (2016).

**Publisher's note:** Springer Nature remains neutral with regard to jurisdictional claims in published maps and institutional affiliations.

## Supplementary Material

Supplementary Table

## Figures and Tables

**Table 1:** Baseline characteristics of study participants with and without CKD (n = 2689).

Characteristics	With CKD (eGFR <60 mL·min^−1.^1.73 m^−2^) (n = 368)	Without CKD (eGFR ≥60 mL·min^−1.^1.73 m^−2^) (n = 2321)	*P*
Age, yr (SD)	73.1 (8.4)	64.1 (8.7)	<0.0001
Male, (%)	132 (35.7)	1041 (44.9)	<0.0001
Receipt of pension	294 (81.4)	1188 (52.2)	<0.0001
Current smoker, (%)	36 (9.9)	328 (14.4)	0.02
Body mass index, kg/m^2^ (SD)	26.4 (4.9)	26.2 (4.3)	0.48
Serum triglycerides, mmol/L (SD)	2.0 (1.1)	1.7 (1.1)	<0.0001
Hypertension^a^ (%)	254 (69.2)	964 (41.6)	<0.0001
Diabetes, (%)	41 (11.2)	158 (6.8)	0.003

eGFR – Estimated glomerular filtration rate (in ml/min/1.73 m^2^) using the 4-variable Modification of Diet in Renal Disease Study formula.

^a^Defined as systolic BP greater than 140 mm Hg or diastolic BP more than 90 mm Hg or using anti-hypertensive medications.

**Table 2:** Association between dairy food intake and prevalence of CKD (stage 3 or greater), presented as adjusted odds ratios (OR) and 95% confidence intervals (CI) among Blue Mountains Eye Study participants in 1992–4 (n = 2689).

Dairy consumption, *servings*/*day*	eGFR <60 mL·min^−1.^1.73 m^−2^		
	No. of cases/no. at risk	Age-sex adjusted OR (95% CI)	Multivariable-adjusted OR (95% CI)
Total dairy^a^			
1^st^ quintile (≤0.75)	83/532	1.0 (reference)	1.0 (reference)
2^nd^ quintile (0.76–1.18)	85/543	0.97 (0.68–1.39)	0.91 (0.63–1.32)
3^rd^ quintile (1.18–1.63)	74/539	0.93 (0.64–1.34)	0.87 (0.60–1.28)
4^th^ quintile (1.64–2.71)	57/538	**0.65 (0.44–0.96)**	**0.64 (0.43–0.96)**
5^th^ quintile (≥2.72)	69/538	0.80 (0.56–1.17)	0.82 (0.56–1.20)
*P* for trend		0.09	0.17
Reduced/low fat dairy^a,b^			
1^st^ quintile (≤0.00)	127/730	1.0 (reference)	1.0 (reference)
2^nd^ quintile (0.02–0.05)	31/351	**0.59 (0.37–0.92)**	0.66 (0.41–1.05)
3^rd^ quintile (0.06–0.57)	69/536	0.86 (0.61–1.22)	0.84 (0.58–1.21)
4^th^ quintile (0.58–1.12)	84/534	1.04 (0.73–1.47)	1.05 (0.73–1.51)
5^th^ quintile (≥1.13)	57/538	**0.65 (0.44–0.95)**	**0.64 (0.43–0.96)**
*P* for trend		0.23	0.23
Regular fat dairy ^a,c^			
1^st^ quintile (≤0.17)	81/533	1.0 (reference)	1.0 (reference)
2^nd^ quintile (0.18–0.47)	71/538	0.94 (0.65–1.36)	0.95 (0.65–1.40)
3^rd^ quintile (0.47–0.90)	70/543	0.80 (0.55–1.16)	0.83 (0.57–1.22)
4^th^ quintile (0.91–1.54)	83/544	0.90 (0.61–1.32)	0.84 (0.56–1.26)
5^th^ quintile (≥1.55)	63/531	0.70 (0.47–1.04)	0.74 (0.49–1.11)
*P* for trend		0.10	0.16

*Further adjusted for receipt of pension, body mass index, smoking, serum triglycerides, hypertension, and history of diagnosed diabetes.

^†^Further adjusted for baseline regular-fat dairy product consumption.

^‡^Further adjusted for baseline low-fat dairy product consumption.

**Table 3:** Association between dairy food intake and 10-year incidence of CKD (stage 3 or greater), presented as adjusted odds ratios (OR) and 95% confidence intervals (CI) among Blue Mountains Eye Study participants in 1992–4 to 2002–4 (n = 1185).

Dairy consumption, *servings*/*day*	eGFR <60 mL·min^−1.^1.73 m^−2^		
	No. of cases/no. at risk	Age-sex adjusted OR (95% CI)	Multivariable-adjusted OR (95% CI)
Total dairy^a^			
1^st^ quintile (≤0.81)	29/237	1.0 (reference)	1.0 (reference)
2^nd^ quintile (0.82–1.22)	38/237	1.33 (0.78–2.28)	1.35 (0.78–2.35)
3^rd^ quintile (1.23–1.76)	48/237	1.73 (1.03–2.91)	1.85 (1.08–3.15)
4^th^ quintile (1.77–2.81)	35/237	1.14 (0.66–1.97)	1.11 (0.63–1.95)
5^th^ quintile (≥2.82)	28/237	0.86 (0.49–1.52)	0.94 (0.52–1.68)
*P* for trend		0.25	0.38
Reduced/low fat dairya,^b^			
1^st^ quintile (≤0.00)	54/259	1.0 (reference)	1.0 (reference)
2^nd^ quintile (0.02–0.09)	23/214	**0.48 (0.28–0.84)**	**0.51 (0.29–0.89)**
3^rd^ quintile (0.09–0.85)	33/238	0.65 (0.39–1.08)	0.64 (0.38–1.09)
4^th^ quintile (0.86–1.22)	32/235	0.60 (0.36–1.01)	0.66 (0.39–1.13)
5^th^ quintile (≥1.24)	36/239	0.64 (0.38–1.07)	0.66 (0.39–1.12)
*P* for trend		0.33	0.39
Regular fat dairya,^c^			
1^st^ quintile (≤0.21)	38/237	1.0 (reference)	1.0 (reference)
2^nd^ quintile (0.22–0.50)	29/236	0.74 (0.43–1.28)	0.76 (0.44–1.32)
3^rd^ quintile (0.52–0.91)	33/238	0.84 (0.49–1.42)	0.78 (0.45–1.34)
4^th^ quintile (0.92–1.55)	40/237	1.00 (0.59–1.69)	0.98 (0.57–1.69)
5^th^ quintile (≥1.56)	38/237	0.93 (0.54–1.58)	0.94 (0.55–1.63)
*P* for trend		0.44	0.44

^a^Further adjusted for receipt of pension, body mass index, smoking, serum triglycerides, hypertension, and history of diagnosed diabetes.

^b^Further adjusted for baseline regular-fat dairy product consumption.

^c^Further adjusted for baseline low-fat dairy product consumption.

**Table 4:** Cross-sectional association between total dietary calcium intake and prevalence of CKD (stage 3 or greater), presented as adjusted odds ratios (OR) and 95% confidence intervals (CI) among Blue Mountains Eye Study participants (1992–4).

Total calcium intake, *mg*/*day*	eGFR <60 mL·min^−1.^1.73 m^−2^		
	No. of cases/no. at risk	Age-sex adjusted OR (95% CI)	Multivariable-adjusted OR (95% CI)
Total calcium intake^a^			
1^st^ quintile (≤543.1)	89/537	1.0 (reference)	1.0 (reference)
2^nd^ quintile (543.2–726.7)	86/537	0.90 (0.63–1.28)	0.81 (0.56–1.17)
3^rd^ quintile (727.1–918.6)	63/537	**0.63 (0.43–0.91)**	**0.57 (0.38–0.84)**
4^th^ quintile (918.7–1225)	67/537	**0.69 (0.47–0.99)**	**0.68 (0.47–1.00)**
5^th^ quintile (≥1226)	63/537	**0.63 (0.43–0.91)**	**0.62 (0.42–0.92)**
*P* for trend		0.01	0.02

^a^Further adjusted for receipt of pension, body mass index, smoking, serum triglycerides, hypertension, and history of diagnosed diabetes.

## References

[b1] Louie J. C. . Higher regular fat dairy consumption is associated with lower incidence of metabolic syndrome but not type 2 diabetes. Nutr Metab Cardiovasc Dis (2012). 10.1016/j.numecd.2012.08.004 23021710

[b2] Malik V. S. . Adolescent dairy product consumption and risk of type 2 diabetes in middle-aged women. Am J Clin Nutr 94, 854–861 (2011). 21753066 10.3945/ajcn.110.009621 PMC3155931

[b3] Lamarche B. Review of the effect of dairy products on non-lipid risk factors for cardiovascular disease. J Am Coll Nutr 27, 741S–746S (2008). 19155434 10.1080/07315724.2008.10719752

[b4] van Meijl L. E. & Mensink R. P. Low-fat dairy consumption reduces systolic blood pressure, but does not improve other metabolic risk parameters in overweight and obese subjects. Nutr Metab Cardiovasc Dis 21, 355–361 (2011). 20153619 10.1016/j.numecd.2009.10.008

[b5] Sanders K. M. . Calcium and bone health: position statement for the Australian and New Zealand Bone and Mineral Society, Osteoporosis Australia and the Endocrine Society of Australia. Med J Aust 190, 316–320 (2009). 19296813 10.5694/j.1326-5377.2009.tb02421.x

[b6] Gopinath B. . Consumption of dairy products and the 15-year incidence of age-related macular degeneration. Br J Nutr 111, 1673–1679 (2014). 24502821 10.1017/S000711451300408X

[b7] Gopinath B., Flood V. M., Wang J. J., Burlutsky G. & Mitchell P. Lower dairy products and calcium intake is associated with adverse retinal vascular changes in older adults. Nutr. Metab Cardiovasc. Dis. 2013. 10.1016/j.numecd.2013.06.009 24418384

[b8] da Silva F. T., Torres M. R. & Sanjuliani A. F. Dietary calcium intake is associated with adiposity, metabolic profile, inflammatory state and blood pressure, but not with erythrocyte intracellular calcium and endothelial function in healthy pre-menopausal women. Br J Nutr 1–10 (2013). 10.1017/S0007114513000111 23411109

[b9] Lauretani F. . Omega-3 and renal function in older adults. Curr Pharm Des 15, 4149–4156 (2009). 20041816 10.2174/138161209789909719 PMC2863302

[b10] Gibson R. A., Makrides M., Smithers L. G., Voevodin M. & Sinclair A. J. The effect of dairy foods on CHD: a systematic review of prospective cohort studies. Br J Nutr 102, 1267–1275 (2009). 19682399 10.1017/S0007114509371664

[b11] Massy Z. A., Stenvinkel P. & Drueke T. B. The role of oxidative stress in chronic kidney disease. Semin Dial 22, 405–408 (2009). 19708991 10.1111/j.1525-139X.2009.00590.x

[b12] Cachofeiro V. . Oxidative stress and inflammation, a link between chronic kidney disease and cardiovascular disease. Kidney Int Suppl S4–S9 (2008). 10.1038/ki.2008.516 19034325

[b13] Rebholz C. M. . DASH (Dietary Approaches to Stop Hypertension) Diet and Risk of Subsequent Kidney Disease. Am J Kidney Dis (2016). 10.1053/j.ajkd.2016.05.019 PMC5123940 27519166

[b14] Gopinath B., Harris D. C., Flood V. M., Burlutsky G. & Mitchell P. A better diet quality is associated with a reduced likelihood of CKD in older adults. Nutr Metab Cardiovasc Dis 23, 937–943 (2013). 22902186 10.1016/j.numecd.2012.07.003

[b15] Gopinath B. . Carbohydrate nutrition is associated with the 5-year incidence of chronic kidney disease. J Nutr 141, 433–439 (2011). 21228263 10.3945/jn.110.134304

[b16] Gopinath B., Harris D. C., Flood V. M., Burlutsky G. & Mitchell P. Consumption of long-chain n-3 PUFA, alpha-linolenic acid and fish is associated with the prevalence of chronic kidney disease. Br J Nutr 105, 1361–1368 (2011). 21255476 10.1017/S0007114510005040

[b17] Kao M. P., Ang D. S., Pall A. & Struthers A. D. Oxidative stress in renal dysfunction: mechanisms, clinical sequelae and therapeutic options. J Hum Hypertens 24, 1–8 (2010). 19727125 10.1038/jhh.2009.70

[b18] Bello A. K., Nwankwo E. & El Nahas A. M. Prevention of chronic kidney disease: a global challenge. Kidney Int Suppl S11–S17 (2005). 10.1111/j.1523-1755.2005.09802.x 16108964

[b19] Attebo K., Mitchell P. & Smith W. Visual acuity and the causes of visual loss in Australia. The Blue Mountains Eye Study. Ophthalmology 103, 357–364 (1996). 8600410 10.1016/s0161-6420(96)30684-2

[b20] Willett W. C. . The use of a self-administered questionnaire to assess diet four years in the past. Am J Epidemiol 127, 188–199 (1988). 3337073 10.1093/oxfordjournals.aje.a114780

[b21] Barclay A. W., Flood V. M., Brand-Miller J. C. & Mitchell P. Validity of carbohydrate, glycaemic index and glycaemic load data obtained using a semi-quantitative food-frequency questionnaire. Public Health Nutr 11, 573–580 (2008). 17956640 10.1017/S1368980007001103

[b22] Smith W., Mitchell P., Reay E. M., Webb K. & Harvey P. W. Validity and reproducibility of a self-administered food frequency questionnaire in older people. Aust N Z J Public Health 22, 456–463 (1998). 9659773 10.1111/j.1467-842x.1998.tb01414.x

[b23] Department of Community Services and Health. NUTTAB90 nutrient data table for use in Australia. Australian Government Publishing Service, Canberra (1990).

[b24] Department of Community Services and Health. NUTTAB95 nutrient data table for use in Australia. Australian Government Publishing Service, Canberra (1995).

[b25] Eat for Health: Australian Dietary Guidelines-Summary. NHMRC. Canberra, Commonwealth of Australia 2013.

[b26] Levey A. S. . A more accurate method to estimate glomerular filtration rate from serum creatinine: a new prediction equation. Modification of Diet in Renal Disease Study Group. Ann Intern Med 130, 461–470 (1999). 10075613 10.7326/0003-4819-130-6-199903160-00002

[b27] Whitworth J. A. World Health Organization, International Society of Hypertension Writing Group. J Hypertens 21, 1983–1992 (2003). 14597836 10.1097/00004872-200311000-00002

[b28] Zemel M. B., Sun X., Sobhani T. & Wilson B. Effects of dairy compared with soy on oxidative and inflammatory stress in overweight and obese subjects. Am J Clin Nutr 91, 16–22 (2010). 19889829 10.3945/ajcn.2009.28468

[b29] K/DOQI clinical practice guidelines for chronic kidney disease: evaluation, classification, and stratification. Am J Kidney Dis 39, S1–266 (2002). 11904577

[b30] Gansevoort R. T., Nauta F. L. & Bakker S. J. Albuminuria: all you need to predict outcomes in chronic kidney disease? Curr Opin Nephrol Hypertens 19, 513–518 (2010). 20802333 10.1097/MNH.0b013e32833e4ce1

[b31] Nettleton J. A., Steffen L. M., Palmas W., Burke G. L. & Jacobs D. R. Jr. Associations between microalbuminuria and animal foods, plant foods, and dietary patterns in the Multiethnic Study of Atherosclerosis. Am J Clin Nutr 87, 1825–1836 (2008). 18541574 10.1093/ajcn/87.6.1825 PMC2503276

[b32] Liu W. C. . Pleiotropic effects of vitamin D in chronic kidney disease. Clin Chim Acta 453, 1–12 (2016). 26656443 10.1016/j.cca.2015.11.029

[b33] Daly R. M. . Prevalence of vitamin D deficiency and its determinants in Australian adults aged 25 years and older: a national, population-based study. Clin Endocrinol (Oxf) 77, 26–35 (2012). 22168576 10.1111/j.1365-2265.2011.04320.x

[b34] Zemel M. B. & Sun X. Dietary calcium and dairy products modulate oxidative and inflammatory stress in mice and humans. J Nutr 138, 1047–1052 (2008). 18492832 10.1093/jn/138.6.1047

[b35] Edwards M. S. . Associations between retinal microvascular abnormalities and declining renal function in the elderly population: the Cardiovascular Health Study. Am J Kidney Dis 46, 214–224 (2005). 16112039 10.1053/j.ajkd.2005.05.005

