# Supplementary material for: Associations between dairy food consumption and chronic kidney disease in older adults
Source: Sci Rep. 2016 Dec 20;6:39532. doi: 10.1038/srep39532 (PMC5171808; doi:10.1038/srep39532)
Supplement: Supplementary Table [file srep39532-s1.pdf]

Associations between dairy food consumption and chronic kidney disease in older adults

Running title: Dairy food intake and chronic kidney disease

Bamini Gopinath[1]

David C Harris[2]

Victoria M Flood[3]

George Burlutsky[1]

Paul Mitchell[1]

[1]Centre for Vision Research, The Westmead Institute for Medical Research, University of Sydney, NSW, Australia.

[2]Centre for Transplantation and Renal Research, The Westmead Institute for Medical Research, University of Sydney, NSW, Australia.

[3]Faculty of Health Sciences, University of Sydney and St Vincent's Hospital, Australia.

Online Supplementary Table 1. Baseline characteristics of study participants who survived or died during the 10-year period (n=2682) [a]

Characteristics	Survivors (n=1952)	Died (n=730)	*P*
Age, yr (SD)	62.7 (7.7)	72.3 (9.1)	<0.0001
Male, (%)	774 (39.6)	398 (54.5)	<0.0001
Receipt of pension	926 (48.4)	552 (77.0)	<0.0001
Current smoker, (%)	247 (12.8)	116 (16.3)	0.02
Body mass index, kg/m^2 (SD)	26.4 (4.4)	25.7 (4.5)	0.0002
Serum triglycerides, mmol/L (SD)	1.7 (1.0)	1.8 (1.2)	0.34
Hypertension, [b] (%)	806 (41.3)	409 (56.0)	<0.0001
Diabetes, (%)	122 (6.3)	77 (10.6)	0.0001
Total dairy food intake	1.7 (1.2)	1.6 (1.2)	0.01
Reduced/low fat dairy intake	0.8 (1.0)	0.6 (1.0)	<0.0001
Regular fat dairy intake	1.0 (0.9)	1.0 (0.9)	0.21
Total dietary calcium intake	906.5 (417)	863.2 (418)	0.02

[a] Seven people had missing mortality data and so were not included in this Table.
[b] Defined as systolic BP greater than 140mm Hg or diastolic BP more than 90 mm Hg or using anti-hypertensive medications.
